# The glycosylation in SARS-CoV-2 and its receptor ACE2

**DOI:** 10.1038/s41392-021-00809-8

**Published:** 2021-11-15

**Authors:** Yanqiu Gong, Suideng Qin, Lunzhi Dai, Zhixin Tian

**Affiliations:** 1 National Clinical Research Center for Geriatrics and Department of General Practice, State Key Laboratory of Biotherapy, West China Hospital, Sichuan University, and Collaborative Innovation Center of Biotherapy, 610041 Chengdu, China; 2 School of Chemical Science & Engineering, Shanghai Key Laboratory of Chemical Assessment and Sustainability, Tongji University, 200092 Shanghai, China

**Keywords:** Infectious diseases, Biological techniques

## Abstract

Coronavirus disease 2019 (COVID-19), a highly infectious disease caused by severe acute respiratory syndrome coronavirus 2 (SARS-CoV-2), has infected more than 235 million individuals and led to more than 4.8 million deaths worldwide as of October 5 2021. Cryo-electron microscopy and topology show that the SARS-CoV-2 genome encodes lots of highly glycosylated proteins, such as spike (*S*), envelope (*E*), membrane (*M*), and ORF3a proteins, which are responsible for host recognition, penetration, binding, recycling and pathogenesis. Here we reviewed the detections, substrates, biological functions of the glycosylation in SARS-CoV-2 proteins as well as the human receptor ACE2, and also summarized the approved and undergoing SARS-CoV-2 therapeutics associated with glycosylation. This review may not only broad the understanding of viral glycobiology, but also provide key clues for the development of new preventive and therapeutic methodologies against SARS-CoV-2 and its variants.

## Introduction

The severe acute respiratory syndrome coronavirus 2 (SARS-CoV-2) is the virus causing the coronavirus disease 2019 (COVID-19), which threatens human health and public safety.^1–5^ SARS-CoV-2 virus is genetically closely related to SARS-CoV,^6–8^ less deadly but far more transmissible.^9–11^ It usually causes a lower respiratory tract infection, and the most common symptoms include fever, malaise, dry cough and shortness of breath, which can progress to severe acute respiratory syndrome and even multiple organ failure.^12–17^ Epidemiology data show that the SARS-CoV-2 pandemic has resulted in more than 235 million confirmed infected cases and more than 4.8 million deaths worldwide as of October 5 2021 (https://covid19.who.int/), urgently calling for effective prevention and intervention therapeutics.^18–20^ In-depth studies on viral infection and pathogenic mechanisms will help to find potential cures for COVID-19.^21–24^

Protein glycosylation is a process of post-translational or co-translational covalent attachment of glycans to the amino acid side chains of proteins.^25–27^ Glycans, being linear or branched chains of monosaccharides, often have high solubility and conformational entropy, which regulates the protein folding, structures, and functions.^28–32^ The SARS-CoV-2 is decorated by a large number of highly glycosylated proteins,^33^ and its glycosylation (both N-linked and O-linked) extensively affects host recognition,^32,34,35^ penetration,^36^ binding,^2^ recycling,^37^ and pathogenesis.^38–42^ In this review, we have systematically introduced the methods for characterizing protein glycosylation, summarized the reported glycosylome of SARS-CoV-2 proteins and its receptor protein ACE2, described the potential biological functions of the glycosylation in SARS-CoV-2, and presented the approved and potential SARS-CoV-2 prevention and treatment theraputics associated with glycosylation.

### Overview of protein glycosylation

Glycosylation is the most common protein post-translational modification (PTM) in virus.^43–47^ Glycosylation not only promotes viral protein folding and subsequent trafficking,^45,48,49^ but also modulates their interactions with receptors and the following innate and adaptive immune response,^50–52^ which affects the host recognition, viral replication, and infectivity.^53–55^ The viruses choose the host cell biosynthetic pathway to produce their genetic and structural materials, and thus the glycosylation of viral proteins greatly depends on the host organelles and enzymes.^45,47,56,57^ As the evolution of viruses, their glycosylome changes, which may cause huge impacts on the survival and transmissibility of the viruses.^45^

**Fig. 1:**
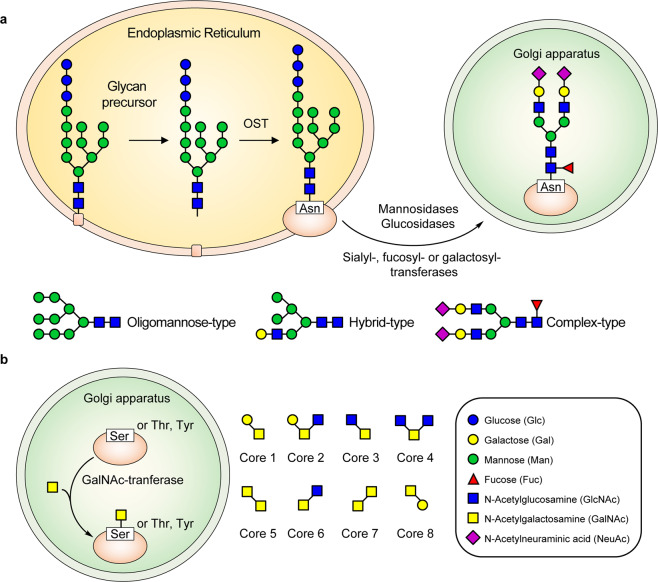
The formation process of N-glycosylation and O-glycosylation in SARS-CoV-2. According to the complexity of the glycans, the N-glycosylation (**a**) is classified into oligomannose-type (2HexNAc), hybrid-type (3HexNAc), and complex-type (with more than 3HexNAc) glycans, whereas the mucin O-glycosylation (**b**) is classified into 8 Core types

N-glycosylation refers to the glycans attached to asparagine (Asn) residue.^43,58^ The glycan precursor (Glc3Man9GlcNAc2) containing three glucose (Glc), nine mannose (Man), and two N-acetylglucosamine (GlcNAc) is first synthesized in the membrane of endoplasmatic reticulum (ER).^59,60^ Then, the glycan precursor is transported to the ER lumen for processing by adding monosaccharides.^61^ When the glycan is matured, it is added to Asn residue by the oligosaccharyltransferase (OST), and the nascent protein is formed.^47^ Next, other enzymes like mannosidases, glucosidases, sialyl-, fucosyl-, or galactosyl-transferases located at the ER-Golgi apparatus decorate the protein.^50,61^ N-linked glycans mainly simplifies into three types based on the structures, including oligomannose (2HexNAc), hybrid (3HexNAc), and complex-type (with more than 3HexNAc) N-glycan structures^62,63^ (Fig. 1a).

O-glycosylation usually occurs on serine (Ser), threonine (Thr), and tyrosine (Tyr) residues,^43,64^ and mucin-type O-glycosylation (N-acetylgalactosamine (GalNAc)-type) is most common in virus.^29,65,66^ In the O-glycosylation process, GalNAc monosaccharide is first transferred by GalNAc-transferases to Ser, Thr, or Tyr residue in the Golgi apparatus.^56,60^ The glycosyltransferases then decorate the O-linked glycans, of which eight core structures have been described (Fig. 1b). Core 1-4 are four common O-GalNAc glycan core structures in mammals,^67,68^ while Core 1 and Core 2 prefer to exist in virus.^69–71^

### Methods to characterize protein glycosylation

Mass spectrometry (MS)-based N-glycoproteomics has been widely adopted for both site- and structure-specific characterization of glycosylation.^2,12,72–80^ Sample preparation, chromatographic separation, LC-MS/MS analysis and bioinformatics data analysis are the four key pipeline steps.^81–92^

**Fig. 2:**
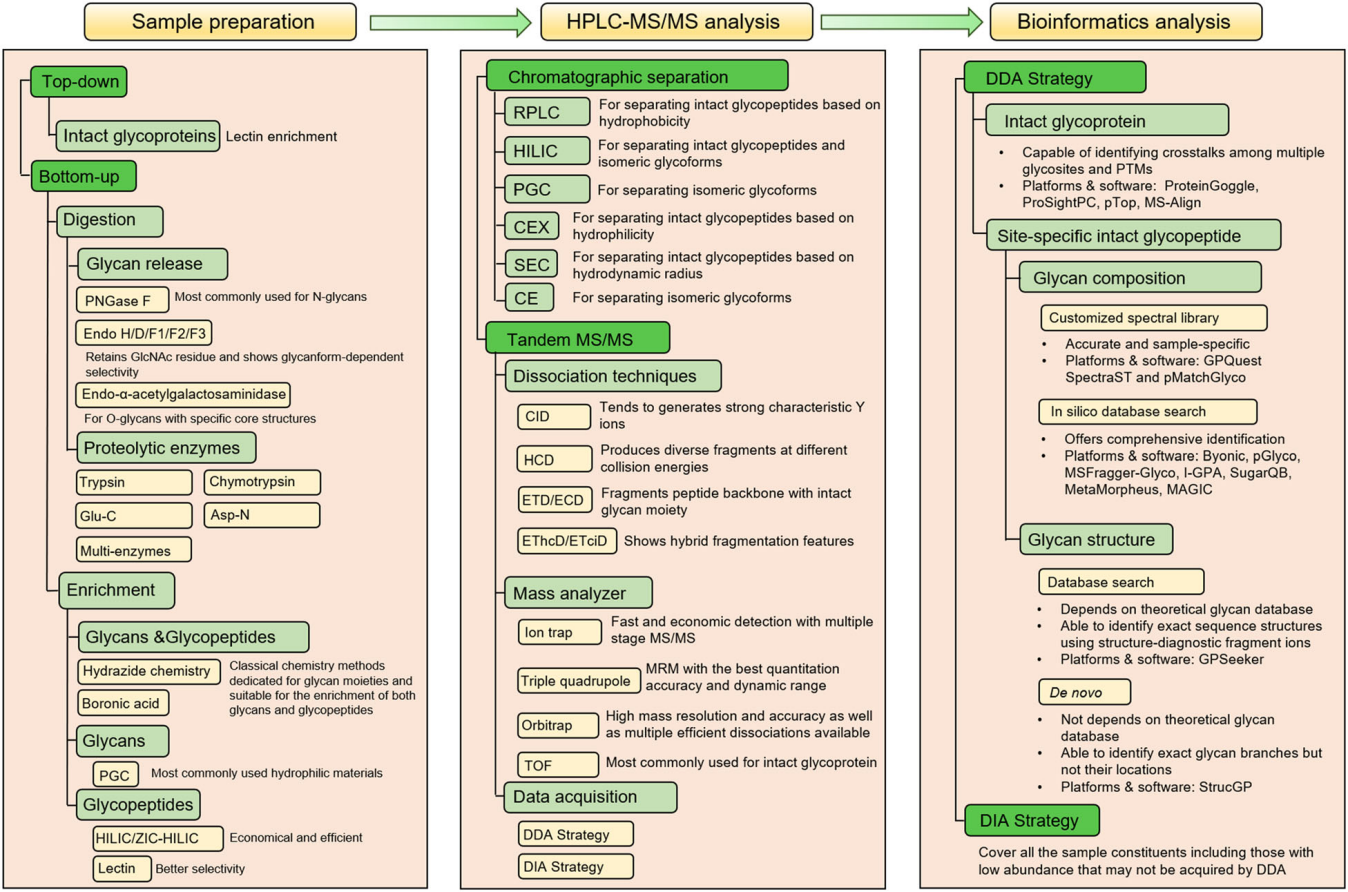
Strategies for mass spectrometry analysis of protein glycosylation

Sample preparation (Fig. 2, left). Glycosylation analysis usually includes the characterization of glycan,^93–95^ intact glycopeptide,^96–98^ glycosite-containing peptide,^99,100^ as well as intact glycoprotein.^101–105^ During the sample preparation, glycan releasing enzymes (such as PNGase F for N-glycans),^106–111^ protease (such as trypsin),^112–117^ both glycan releasing and protease,^118,119^ or no enzymatic approaches may be adopted.^120–123^ Glycans usually need to be enriched by hydrophilic materials, such as porous graphitized carbon (PGC),^124,125^ before MS analysis,^126–128^ while intact glycopeptides can be analyzed by MS with or without enrichment,^129^ although the enrichment step is beneficial for deep characterization of glycopeptides with low stoichiometry.^130–145^ Historically, chemical enrichment methods were adopted for both glycans and glycopeptides such as hydrazide chemistry,^146–149^ boronic acid,^142,150–152^ etc. Hydrophilic interaction liquid chromatography (HILIC),^100,153–155^ lectin affinity chromatography,^121,156,157^ and graphitized carbon chromatography are the most widely adopted methods for enrichment of glycopeptides.^124,158–161^

LC-MS/MS analysis (Fig. 2, middle). Before tandem MS/MS analysis, chromatographic separation can simplify the composition of glycans and glycopeptides.^162^ The underivatized native glycans are hydrophilic and usually separated by PGC columns,^163–165^ while the permethylated glycans are hydrophobic and often separated by reversed-phase C18 chromatography.^166,167^ For the separation of glycosite-containing peptides and intact glycopeptides, the reversed-phase C18 chromatography,^166,168–175^ HILIC,^176–178^ and PGC are also widely used.^179,180^ Moreover, cation-exchange chromatography (CEX),^181,182^ size-exclusion chromatography (SEC),^183–185^ and capillary electrophoresis (CE) are also applied to the separation step.^186–188^

**Fig. 3:**
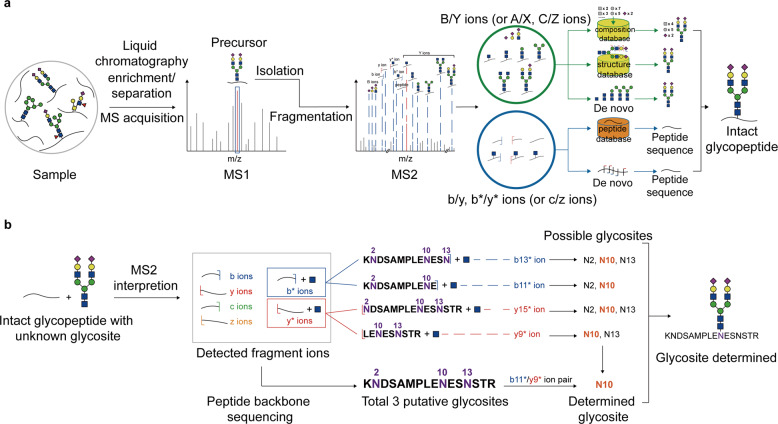
The general workflow of the characterization of intact glycopeptide using MS/MS (**a**), and the algorithm of N-glycosite determination using paired b*/y* ions detected in MS2 spectra (**b**)

The separated glycans or glycopeptides are then analyzed by tandem MS/MS with various dissociation methods^97,189–192^ (Fig. 2, middle). Because of the possible appearance of multiple putative glycosites on a single peptide and the frequent presence of structural isomers in glycans,^165,193^ glycosite localization and glycan structure identification are the two major challenges in MS/MS analysis of both N- and O- glycosylation. A general workflow for the MS/MS analysis of intact glycopeptides are shown in Fig. 3 (note that this workflow is a stereotyped summarize of schemes for the identification of intact glycopeptides, which means many studies will not strictly follow this workflow). The N-glycosites can be localized by site-determining fragment ions from MS2 spectra,^194^ and structural isomers are distinguished with structure-diagnostic fragment ions of the N-glycan moieties.^195^ While O-glycosites, due to the frequent existence of three target amino acids (S/T/Y) and densely glycosylated adjacent sites, are much more difficult to be determined than N-glycosites.^196–203^

N-glycosites rigorously follow the motif rule of N-X-S/T/C (X represents any amino acid except proline),^204,205^ and several other peculiar motif rules have been reported.^99,206–208^ Therefore, the methods for acquiring evident MS2 signals and the algorithms of parsing the MS2 data are necessary.^209–213^

A precise site-determination method for N-glycosites is to use paired b*/y* ions in the MS2 spectrum,^214^ where b* and y* ions respectively refer to peptide b and y ions with a connected GlcNAc residue^215^ (Fig. 3b). These b* and y* ions often appear with moderate abundance after the cleavage of glycopeptide precursor ions.^216^ Several detected b* or y* ions can narrow the possible area of N-glycan moiety and make it covers only one putative site so that the real N-glycosite can be determined.^72,217,218^

Collision-based dissociation, such as collision induced dissociation (CID) and higher energy collisional dissociation (HCD),^219^ can cause peptide fragmentation (either glycosite-containing peptides or intact glycopeptides) and produce abundant b/y fragment ions. Electron-based dissociation, such as electron capture dissociation (ECD)^220^ and electron transfer dissociation (ETD),^221^ has the advantage of causing “gentle” dissociation of the peptide backbone without neutral loss of the N-glycan moiety, generating c/z ions. Ultraviolet photodissociation (UVPD),^222^ simultaneously including the features of both collision- and electron-based dissociation, provides comprehensive types of fragment ions. Selective fragmentation of either the peptide backbone or the N-glycan moiety can be achieved with the combination of different dissociation methods (such as ETD + CID) or different energies of the same dissociation method (such as high and low normalized collision energies of HCD).^223–227^ In addition, combinatory dissociation methods such as combined EThcD and ETciD on Orbitrap mass spectrometers have also been applied.^228,229^

N-glycans on N-glycoproteins contain hundreds of compositions and more than ten thousand different structures in mammals.^230^ N-glycosylation occurring on an identical site of glycoprotein may have thoroughly different biological processes because of distinct monosaccharide compositions.^231–242^ Even N-glycans sharing the same monosaccharide composition may have different functions due to the glycan structures,^243,244^ indicating that the significant roles of N-glycan structures in regulating the functions of N-glycoproteins.^245^ Therefore, structure-specific characterization of N-glycans is urgently needed at both aspects of chemistry and biology.^246^ In general, tandem MS/MS analysis of intact N-glycopeptides is able to precisely identify peptide backbone sequences, N-glycosites as well as N-glycan compositions and structures.^247,248^ However, due to the limitation of MS analytical discernibility, some monosaccharide isomers are unable to be distinguished. For example mannose, galactose, and glucose are interpreted as hexoses in glycan compositions.^249^ Moreover, N-glycans with the same monosaccharide composition may as well form different structures with different amount of antenna and serial numbers of linked carbon atoms (β-1,2 or β-1,4 at α-1,3 core mannose, etc.).^87,250,251^

To unambiguously discriminate the structural isomers, a pivotal series of fragment ions in MS2, herein named structure-diagnostic ions are required.^252^ This kind of ions are in fact the fragmented N-glycan A/B/C/X/Y/Z ions which can independently distinguish a specific structure from the structural isomers.^253,254^ N-glycan structures can be discriminate by detecting theoretical structure-diagnostic ions which are generated in silico relying on the theoretical N-glycan structure database created by the retrosynthetic strategy,^78^ and structures of intact N-glycopeptides are figured out by assigning N-glycan structures to peptide backbones.^255^

Bioinformatics analysis (Fig. 2, right). For identifying intact glycopeptides from LC-MS data, two strategies of MS data acquisition have currently been adopted: data-dependent acquisition (DDA) and data-independent acquisition (DIA).^256–258^ Most of the software and platforms for analyzing intact glycopeptides are designed to search against the spectra generated from DDA.^259–280^ DDA focuses on the precursors with high intensity and specifically isolates them to form fragments and generate MS2 spectra.^281–283^ Based on the DDA data, the method for identifying intact N- and O-glycopeptides, which consists of the following steps (the order of these steps may be rearranged in several platforms or software): (1) deducing peptide backbone by peptide fragment ions, (2) determining glycan mass by calculating the mass difference between deduced peptide backbone and intact glycopeptide precursor, (3) localizing glycosite by matching specific glycosite-containing ions, and (4) characterizing glycan composition or structure using glycan or glycan-containing fragment ions, is adopted by most of the software such as Byonic,^284^ pGlyco,^285,286^ GPQuest,^287^ GPSeeker,^252^ O-pair Search in MetaMorpheus,^288^ MSFragger-Glyco^289^, and StrucGP.^248^ In-silico digested theoretical peptide database or customized experimental peptide spectra library is used for the identification of peptide backbone of intact glycopeptide.^290^

The characterization of glycans (especially N-glycans) by DDA can be achieved by several strategies (Fig. 3a), including (1) parsing N-glycan compositions using theoretical glycan composition database (for instance Byonic,^284,291–294^ which calculates the precise masses of glycans constructed by proper combinations of monosaccharides, giving the number of Hex, HexNAc, etc). These compositions together with their masses are then stored in the composition database and the exact masses of relevant theoretical fragment ions are also calculated and matched in the MS2 spectra for further characterization; (2) parsing N-glycan structures using theoretical structure database built by retrosynthesis rules (for instance GPSeeker^72^), and (3) parsing N-glycan structures using de novo algorithm (for instance StrucGP^248^). The first strategy only offers the information of monosaccharide composition, while the second and third strategies can provide N-glycan structure information. In particular, the second strategy uses structure-diagnostic ions to distinguish different theoretical structures from the same monosaccharide composition and provides the entire structure of each characterized N-glycan. In contrast, the third strategy sequentially matches a series of Y ions and complementary B ions to form an intact N-glycan structure (that is, de novo algorithm), and shows structures with high accuracy regardless of theoretical database. However, the third strategy may ambiguously distinguish symmetrical structures in some applications. StrucGP is the first search engine that adopts de novo algorithm to conduct structure-specific identification of intact glycopeptides.^248^

The application of DIA to identify intact glycopeptides is still very young.^295,296^ Compared with the DDA strategy, DIA does not select specific precursors based on MS1 peak intensities.^256^ Instead, DIA collects all ions acquired in MS1 based on retention time and fragments these ions to generate MS2 spectra,^256,295^ suggesting that LC-MS/MS data from DIA contains complete information of the sample rather than DDA data which only contains the information of peptides with high abundance. However, interpreting DIA data remains a challenge and needs more advanced algorithms such as machine learning.^297,298^ The techniques adopted to analyzing DIA data includes pre-building corresponding DDA data library and many other methods.^296,299,300^ As for the identification of intact glycopeptide using DIA strategy, SWATH-MS workflow has also been adopted,^301^ and the characterization of glycosylation has been achieved at the molecular levels of intact glycopeptide and glycan.^302,303^

### Glycosylation of SARS-CoV-2 proteins

The aforementioned high-throughput detection and analysis of the structure and localization of protein glycans is a prerequisite for discovering and studying the function of glycosylation,^28,87,304^ which will lead to a better understanding of glycoprotein functions and the molecular mechanisms of infectious disease.^38,50,305–307^

SARS-CoV-2 is a positive-sense single-stranded RNA virus.^308,309^ Sequence analysis of SARS-CoV-2 isolates shows that the 30 kb genome at least encodes 29 proteins, including 4 structural proteins, 16 non-structural proteins (NSP1-NSP16), and 9 accessory factors (ORF3a, ORF3b, ORF6, ORF7a, ORF7b, ORF8, ORF9b, ORF9c, ORF10).^310,311^ The NSPs involve in virus processing and replication,^312–315^ while the structural proteins including spike (*S*), envelope (*E*), membrane (*M*), and nucleocapsid (*N*) are responsible for host recognition, binding, recycling, and pathogenesis.^34,36–42,316^ According to the in silico topology, the majority of the encoded proteins are glycoproteins, although only four of them have been reported with their exact glycosites to date.

#### S protein

Among the structural proteins, *S* protein in SARS-CoV-2 is the only one with sequence variability >20% when compared with SARS-CoV.^317^ It is a trimeric transmembrane protein that composes of two functional subunits S1 and S2.^318,319^ The S1 subunit is responsible for host cell receptor binding, while S2 subunit is for membrane fusion.^320,321^ The total length of *S* protein is 1273 amino acids, and the receptor binding domain (RBD) is located in the region from amino acid 319 to 541 in S1 subunit.^322^ The receptor binding motif (RBM) that mediates the contact with the angiotensin-converting enzyme 2 (ACE2) receptor locates in the RBD from amino acid 437 to 507.^320,321,323^ The *S* protein can recognize and bind to ACE2 receptor as the primary host cell infection route.^324^ Therefore, *S* protein determines the infectivity and transmissibility of SARS-CoV-2 and is the major antigen and target of vaccination.^325,326^

**Fig. 4:**
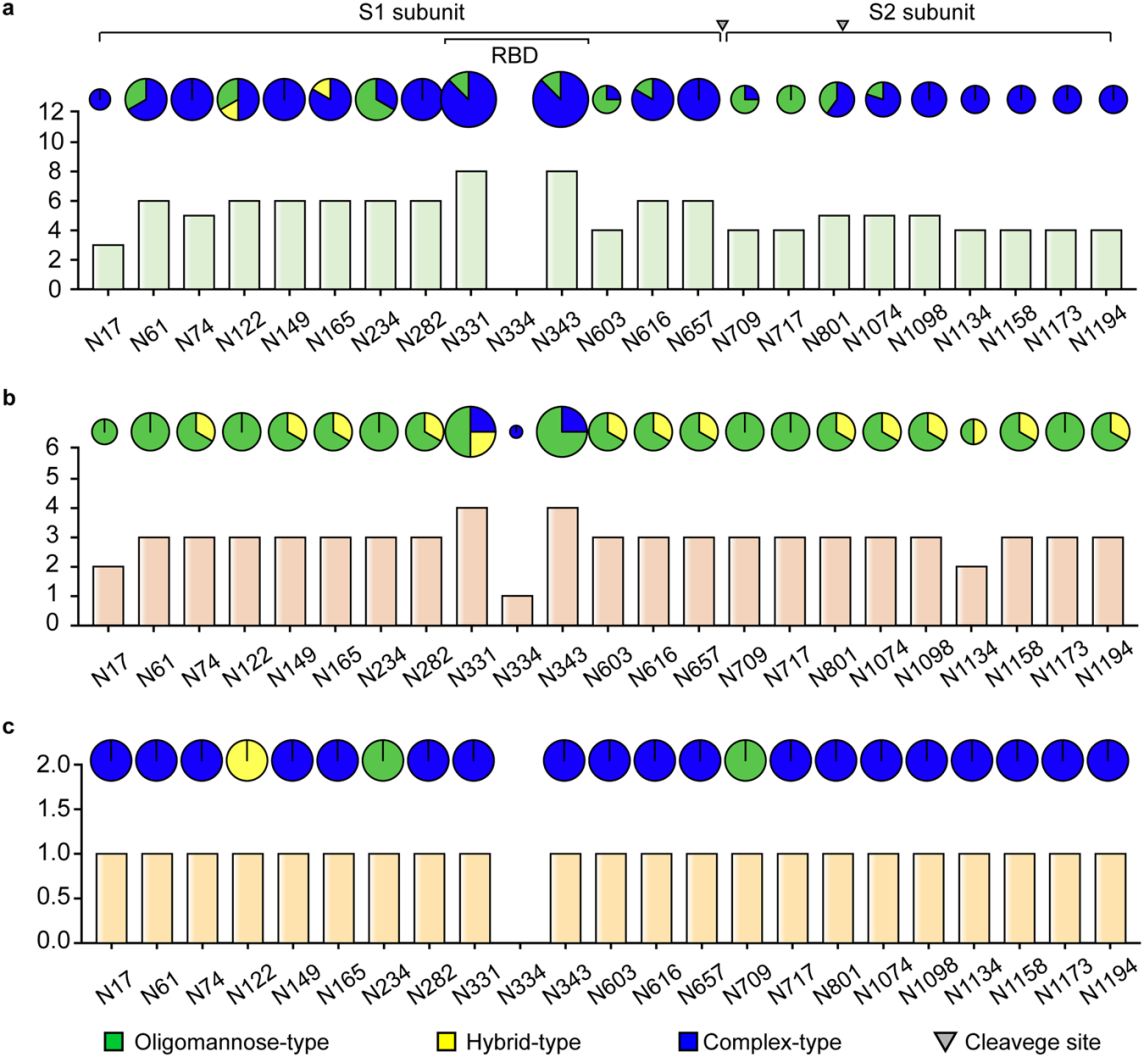
Site-specific N-glycan types of recombinant SARS-CoV-2 *S* proteins expressed in human cells (**a**), insect cells (**b**), or from native *S* protein (**c**). The Y-axis of the histogram refers to the number of published papers that report the corresponding sites with detailed site-specific N-glycan types. The proportion in the pies represents the glycan types reported in the relevant papers. The region of RBD and the furin cleavage site are marked

*S* protein is a well-known glycoprotein, and the modified glycans shield about 40% of the protein surface of the *S* trimer,^35^ which functions as camouflage to humoral and cellular components of the host innate immune system.^54^ Compared with Middle East respiratory syndrome coronavirus (MERS-CoV) and SARS-CoV, the *S* protein of SARS-CoV-2 has a lower glycosylation density,^35,63,327^ indicating the *S* protein surface is more exposed and it is more effective in eliciting humoral immunity.^31^ Since the first report of 16 N-linked glycosites by cryo-electron microscopy (cryo-EM),^320^ the characterization of glycosylation of *S* protein becomes a hotspot.^38,62,63,70,145,328–336^ In total, 23 N-linked glycosites with high occupancy (mostly >95%) have been reported (Fig. 4).^40,62,63,70,328–335^ In contrast, among all the O-linked glycosites, only two sites show relative high occupancy (Table 1).^62,63,70,80,328,332,333,336–342^ The S1 subunit has 13 putative N-glycosites (N17, N61, N74, N122, N149, N165, N234, N282, N331, N343, N603, N616, and N657) with the N-X-S/T (X ≠ P) sequon, one putative N-glycosite (N334) with the N-X-C (X ≠ P) sequon and two putative O-glycosites (T323 and S325), of which T323, S325, N331, N334, and N343 are located on RBD. The S2 subunit has 9 putative N-glycosites (N709, N717, N801, N1074, N1098, N1134, N1158, N1173, and N1194) with the N-X-S/T (X ≠ P) sequon.

**Table 1:** The reported O-glycosites and O-glycopeptides in *S* protein

Expression system	Glycosites and glycopeptides	Description	Refs.
	T323, S325	Low occupancy (<1%)	^63^
	T323, S325	Core-1 and Core-2 O-glycans at T323	^70^
	T678	Core-1 and Core-2 O-glycans, 13% occupancy	^332^
	O-glycopeptides: CDIPIGAGICASYQTQTNSPR, TQLPPAYTNSFTR, YFKNHTSPDVD, VQPTESIVR, VYSTGSNVFQTR, VPVAIHADQLTPTWR, STECSNLLLQYGSFCTQLNR, LPDDFTGCVIAWNSNNLD, YSVLYNSASFSTFK	The glycopeptides are identified, but the glycosites and the glycans are not characterized	^332^
Human cells	T22, T29, S31, T33, T124, T284, T286, S297, T299, T302, S305, T307, T315, S316, T323, S325, T572, T573, T581, T630, T632, S637, T638, S640, T645, S659, S673, T676, T678, S680	Mainly modified with Mucin-type O-glycans	^339^
	T323, S325	Core-1 and Core-2 O-glycans at T323	^328^
	T63, T73, T76, T114, T167, T236, T478, T618, T676, T678, S803, T1076, T1077, S1097	Low overall occupancy	^336^
	T73, S221, T284, T323, S325, T547, T618, T630, T632, S673, T716, S803, S810, S813, S975, S1123, T1136, S1175	Mainly modified with HexNAc(2)Hex(3)	^338^
	T323	Core-1 and Core-2 O-glycans at T323	^340^
Human cells	T22, T29, S31, T33, S50, T51, S71, T73, S94, T95, S98, T108, T109, S247, T250, S254, S255, S256, T259, T286, S297, T299, T315, S316, T323, S325, T333, S349, S359, S366, S371, S375, T376, T430, T470, S477, T478, S494, T500, T523, T547, T553, S555, T581, S591, T599, T630, T632, S637, T638, S640, T645, S673, T676, T678, S680	GalNAc and GalGalNAc mucin-type O-glycans at T323 and T325	^342^
Insect cells	T333, S438, S443, S514, T523, S366, S371, S373, S375, T376	The glycan types are not determined	^333^
	T22, T29, S31, T33, S50, T51, S71, T73, S94, T95, S98, T108, T109, S247, T250, S254, S255, S256, T259, T286, S297, T299, T315, S316, T323, S325, T333, S349, S359, S366, S371, S375, T376, T430, T470, S477, T478, S494, T500, T523, T547, T553, S555, T572, T573, T581, S591, T599, T630, T632, S637, T638, S640, T645, S673, T676, T678, S680	T22, T29, S31, T33, S94, T95, T323 and T325 are the most abundant O-glycosites	^342^
	T22, T29, S31, S94, T95, T114, S116, T124, T284, T286, S297, T299, T323, S325, T333, T345, S477, T572, T573, S659, S673, T676, T678, T732, T791, S803, S810, S813, T912, S939, S940, T941, T1066, T1076, T1077, S1097, T1100, T1105, T1160, S1161, S1170, S1175, S1196	Mucin-type O-glycans	^339^
Insect cells	T63, S71, T73, T76, S94, T95, T124, S151, T315, T323, T333, T345, T415, T523, T678, S803, T1076, T1077, S1097, T1100	Low overall occupancy	^336^
From SARS-CoV-2 virions	T22, S60, T124, S151, T236, S305, T307, T323, T604/S605, T618, S659, T696, T724, T1076, T1077, S1097, T1100	Occurred together with N-glycosites in N-sequon-associated positions	^80^
Chinese hamster ovary cells	T323, S325	Core-1 O-glycans at T323	^341^
Not available	S673, T678, S686	Predicted O-glycosites	^337^
Not available	T323, S325	Core-1 and Core-2 O-glycans	^62^

**Fig. 5:**
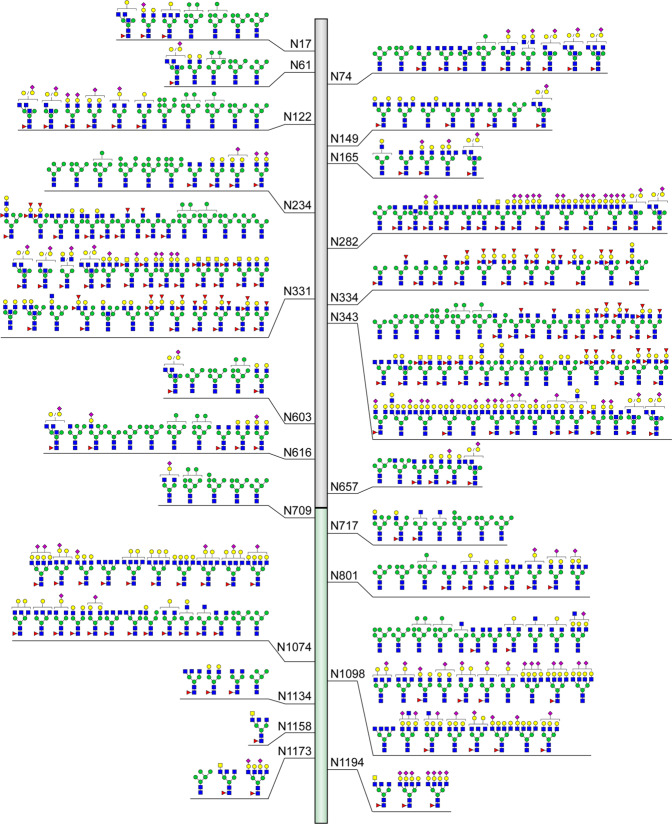
Compiled structures of N-glycans identified on 23 N-glycosites of *S* protein. Studies were summarized only when the reported N-glycans were characterized by fragment ions in MS2 spectra that can help deduce their structures or N-glycans were exhibited with annotations to reveal their exact structures. A little ambiguousness in N-glycan structures such as uncertain position of terminal monosaccharide are allowed. Studies that reported N-glycans with only monosaccharide compositions were not placed in this figure

Although the N-glycosites of *S* protein identified by different teams in different expressed systems are almost same, the glycan compositions and structures as well as their occupancy are distinct (Fig. 4). MS-based characterization of recombinant *S* protein expressed in human cells including human embryonic kidney (HEK) 293F cells and HEK 293 cells shows that the glycans on N234 and N709 are mainly oligomannose-type.^32,40,63,70,329^ Complex-type glycans can be predominantly found at N17, N74, N149, N165, N282, N331, N343, N616, N657, N1098, N1134, N1158, N1173, and N1194 residues, while six positions including N61, N122, N603, N717, N801, and N1074 are modified by a mixture of oligomannose- and complex-type glycans.^63^ Notably, the most common oligomannose-type glycan is Man5GlcNAc2. More than half of these N-linked glycans are fucosylated,^63^ and highly processed sialylated complex-type glycans can be predominantly found on the residues of N165, N282, N801, N1074, and N1098^70,332^ (Fig. 5). By using energy-optimized LC–MS/MS method, glycoforms including the LacdiNAc and polyLacNAc structural motifs have been revealed on N-glycans of *S* protein expressed in the HEK293 expression system.^225,332^ Moreover, a recent quantitative N-glycan analysis on protein of S1 subunit purified from SARS-CoV-2 infected Calu-3 cells by immunoaffinity purification showed that the complex-type N-glycans (79%) with 21% oligomannose and/or hybrid structures predominate.^343^ In addition to the diverse N-linked glycans of *S* protein identified by MS, the N-linked glycan structures of RBD of *S* glycoprotein expressed in human HEK293F cells have been characterized by nuclear magnetic resonance (NMR) spectroscopy, which avoids sample digestion and derivatization.^330^ A lot of glycan structures missed in MS-based approaches have been disclosed.^330^ Besides the expected N-acetyllactosamine (LacNAc), 3′SLN (3′SLacNAc), and 6′SLN (6′SLacNAc) terminal moieties at the glycans of N331 and N343, the unprecedented structures such as LeX (LewisX), LDNF (LeX and fucosylated lacdiNAc), and 6′SLDN (6’SLacdiNAc) were also identified.^330^

The distinct types of N-linked glycans of *S* protein in different expression systems are proposed to be determined by the differential processing of N-glycans among different species, rather than the location of glycosites.^62,342^ Different from that in human cell expression system (Fig. 4a),^40,62,63,70,328–330,332^ the N-linked glycans of recombinant *S* protein obtained from insect cell expression system are most high mannose-type^62,329,333,334^ (Fig. 4b). It is worth noting that besides the N331 and N343 in the RBD, glycosylation at N334 has also been detected in recombinant *S* protein expressed from Spodoptera frugiperda (Sf9) cells with low occupancy,^333^ which is consistent with the report of an N-X-C motif exhibiting substantial N-glycosylation^344^ (Fig. 4b). Interestingly, compared with the recombinant *S* protein in insect cells, native *S* protein has lower levels of oligomannose-type glycans and high levels of complex-type glycans^331^ (Fig. 4c). These studies collectively generated a comprehensive N-glycosylation map of the *S* protein, and all the identified structures are plotted in Fig. 5.^35,40,62,63,70,145,328–333,335,336,341–343,345^

**Fig. 6:**
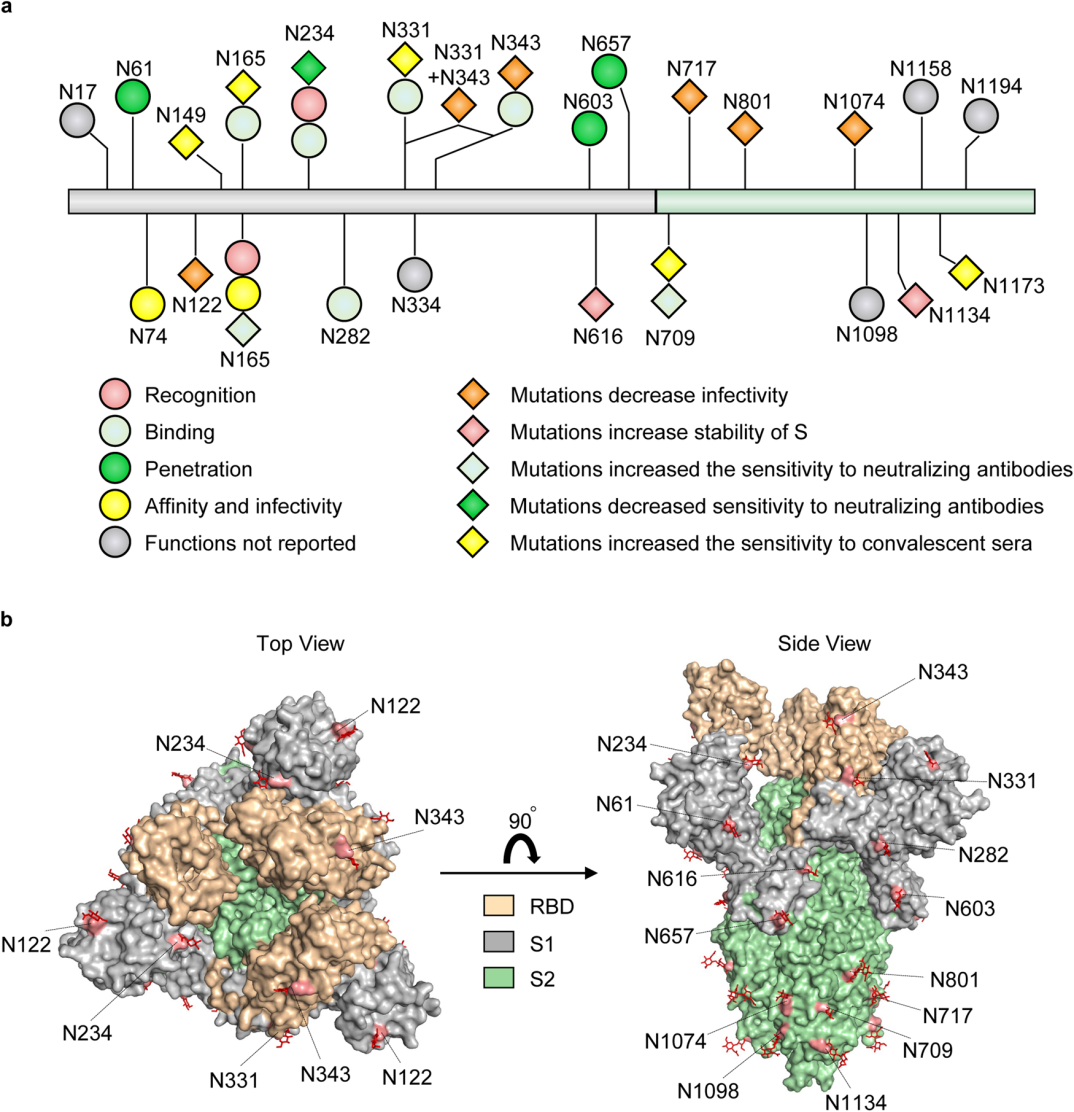
Glycosites of *S* protein and their functions. **a** The functions of glycosylation of *S* protein. Different shapes and colors represent the corresponding functions, and the gray indicates that the functions of the sites are unknown. **b** Structure-based display of the N-glycosites in the *S* protein. The glycosites of *S* protein are marked on the three-dimension structures. *S* protein is shown in the “RBD up” state. A top view and a side view (up) of the *S* protein (PDB: 6VYB) are presented. N74 and N165 are not labeled due to the information missed in the crystal structure^331^

The N-linked glycosites of *S* protein have diverse functions (Fig. 6). N165 and N234 are located near the RBD.^63,346^ N-linked glycosylation at N234 is largely accessible to α-1,2-mannosidases and can regulate the conformational dynamics of RBD.^34^ Deletion of the glycans through N165A and N234A mutations significantly changed the RBD conformational shift towards to the “down” state (presenting a receptor-inaccessible state) and reduced the binding to ACE2, which suggests that the glycosylation at N165 and N234 may promote host recognition.^34^ Moreover, N282, N331, and N343 are also proximal glycosites that shield the receptor binding sites of *S* protein, especially the RBD in the “down” state.^63,70,320,347^ Besides the involvement of these glycosites in the binding of SARS-CoV-2 to the receptor, these glycosites also affect the sensitivity of viruses to neutralizing antibodies. For example, N234Q mutation can significantly decrease the sensitivity to neutralizing antibodies, whereas N165Q and N709Q mutations increase the sensitivity.^2^ In addition, N149Q, N331Q, and N1173Q mutations also dramatically increase the sensitivity to convalescent sera,^39,316,348^ indicating the influence of glycans in epitopes targeted by neutralizing antibodies (Fig. 6). The glycosylation is also important for virus virulence and viral infection. When the glycosylation at N122, N331 with N343, N717, N801, and N1074 of *S* protein are inhibited by mutations, the viral infectivity of SARS-CoV-2 is significantly reduced^39,316^ (Fig. 6). The polybasic cleavage site (RRAR) at the junction of S1 and S2 subunits is one notable feature of SARS-CoV-2, which is not observed in SARS-CoV.^349,350^ The RRAR can be cleaved by furin or other proteases and play important roles in determining viral fusion, entry and pathogenesis.^36,351,352^ The N-glycosylation at N61, N603 and N657 is proximal to the furin-site and able to increase the steric hindrance for cleavage, which seems to be beneficial to SARS-CoV-2 entry^36^ (Fig. 6). Different from being cleaved by proteases, computational saturation mutagenesis of N616 and N1134 residues increases the stability of *S* protein,^353^ which may be associated with glycosylation; however this phenotype needs experimental confirmation (Fig. 6). 3D structural modeling of glycosylated SARS-CoV-2 trimmer *S* protein disclosed the micro-heterogeneity of N-glycosites. The glycans at N74 and N165 residues of *S* protein interact with ACE2 receptor glycan at N546 residue and thus modulate Spike-ACE2 interactions, suggesting that the changes of glycans occupancy may affect the affinity and alter the infectivity^40^ (Fig. 6).

Besides the N-glycosites, a large number of putative O-glycosites have also been found in *S* protein (Table 1). Among them, the levels of O-glycosylation at T323 and S325 are relative higher, while the glycosylation of other O-glycosites are in low occupancy.^40,63,70,329,332,336–338^ The O-glycans identified on O-glycosites of S protein and RBD from human cells as well as deduced O-glycan structures were summarized^70,80,328,332,336,338,340–342,354–357^ (Table 2, Table 3). O-linked glycans such as Core-1,^336^ disialylated Core-1,^332^ Core-2,^328^ mucin-type,^339^ and sialylated mucin type are reported on the recombinant *S* protein.^70^

**Table 2:**
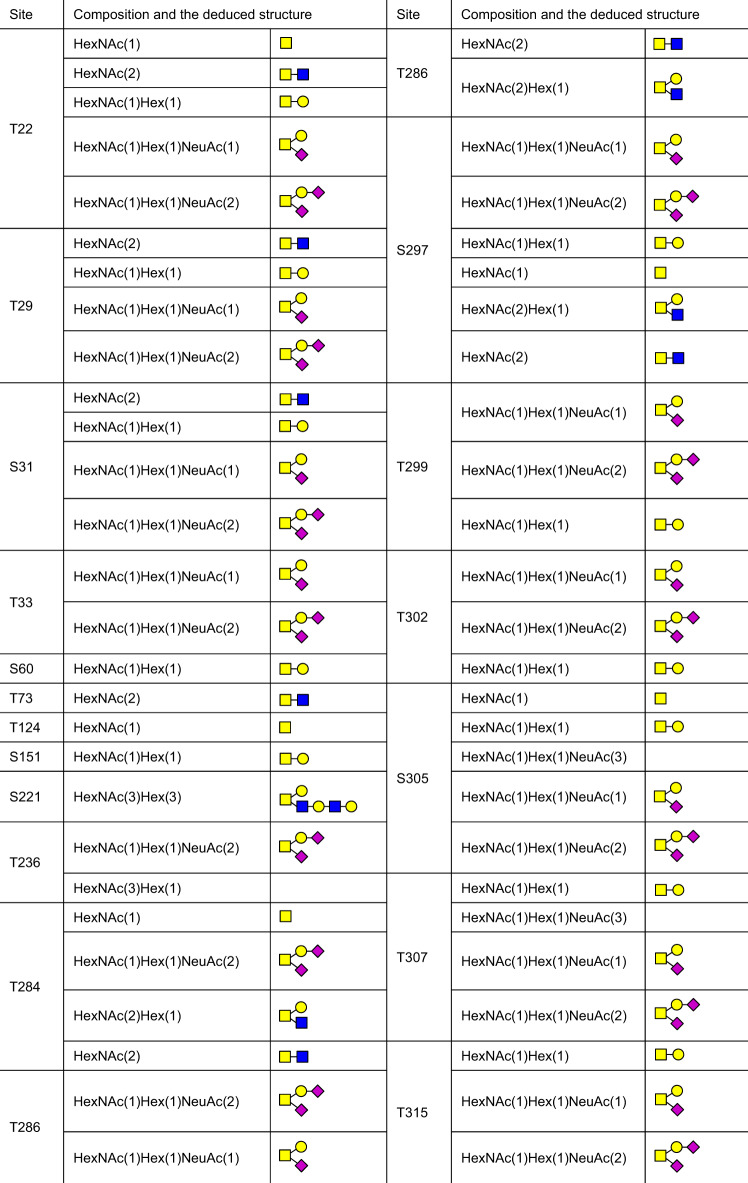

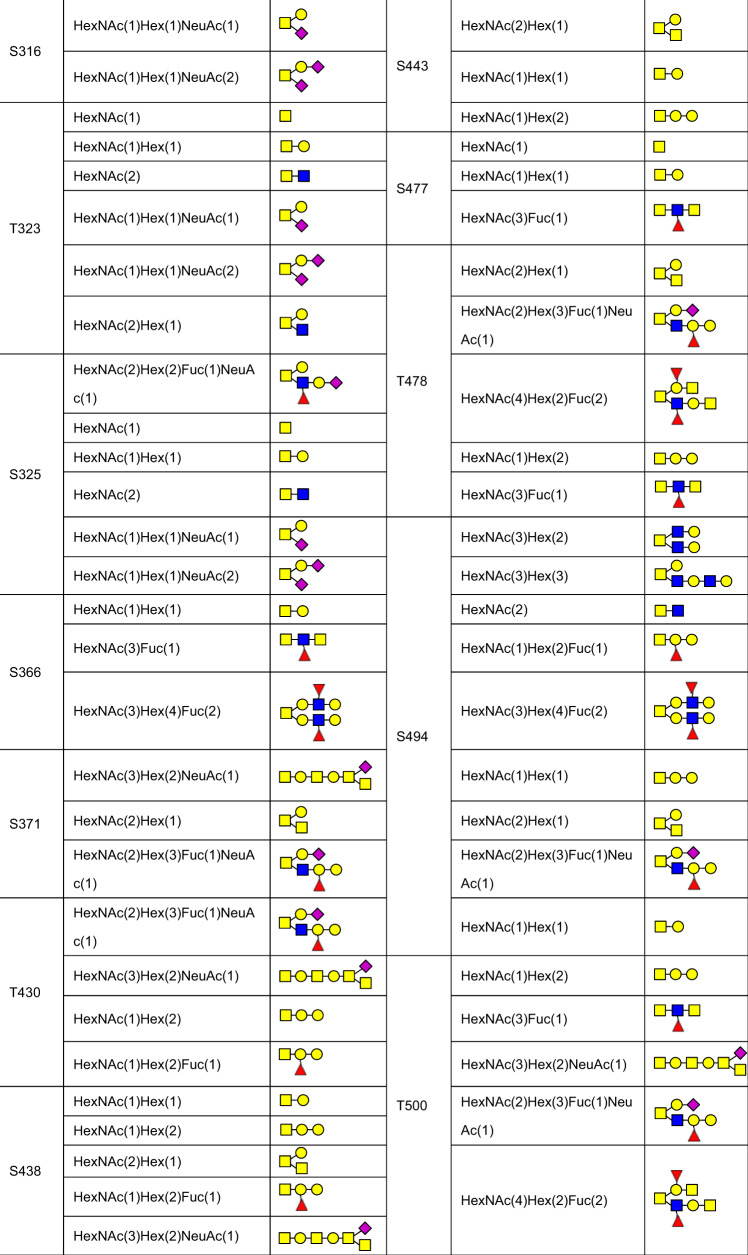

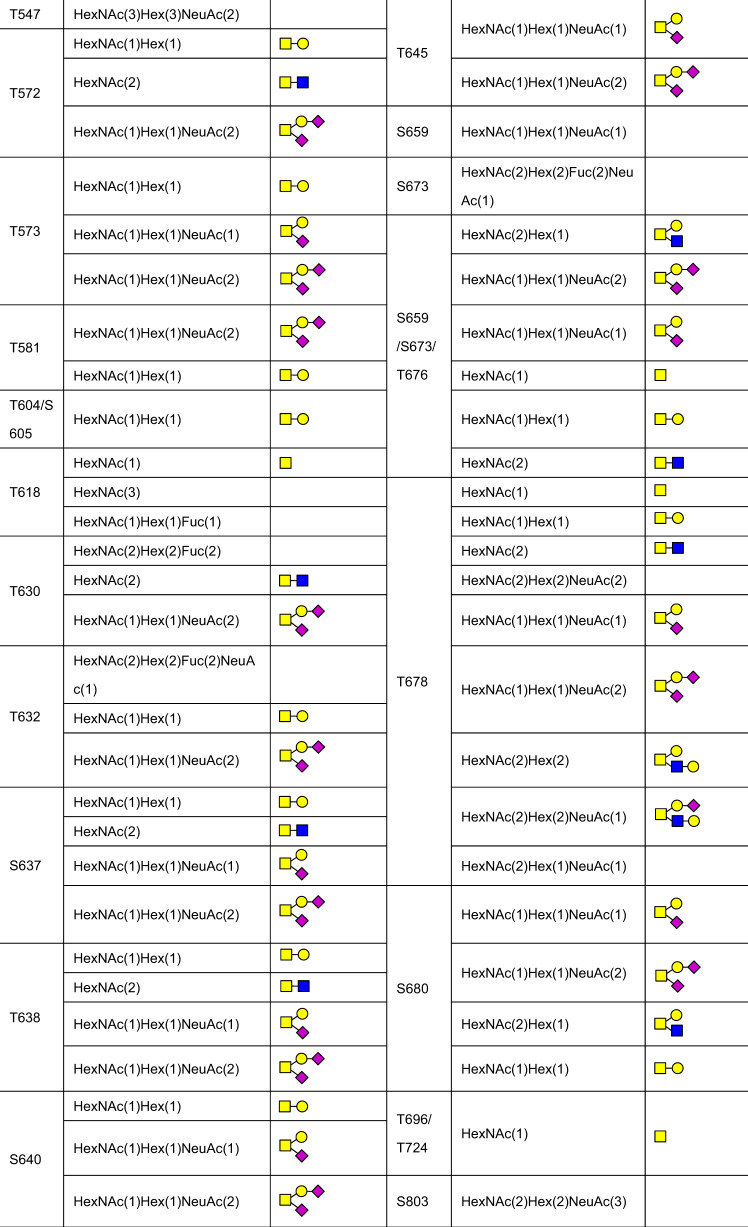

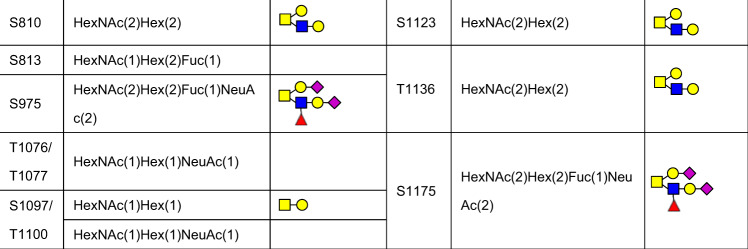
The site-specific assignment as well as deduced O-glycan structures for the O-glycosylation of *S* protein

**Table 3:** The site-specific assignment as well as deduced O-glycan structures for the O-glycosylation of RBD

Composition	Deduced structure	Composition	Deduced structure
HexNAc(1)		HexNAc(2)	
HexNAc(1)Hex(1)		HexNAc(1)NeuAc(1)	
HexNAc(1)Hex(1)NeuAc(1)		HexNAc(2)Hex(1)Fuc(1)	
HexNAc(2)Hex(1)NeuAc(1)		HexNAc(2)Hex(1)	
HexNAc(3)Hex(1)		HexNAc(3)Hex(1)NeuAc(1)	
HexNAc(2)Hex(2)Fuc(2)NeuAc(1)		HexNAc(1)Hex(1)Fuc(1)NeuAc(1)	
HexNAc(2)Hex(2)Fuc(1)NeuAc(1)		HexNAc(3)Hex(1)Fuc(1)NeuAc(1)	
HexNAc(2)Hex(2)		HexNAc(2)Hex(2)NeuAc(2)	
HexNAc(2)Hex(1)NeuAc(2)		HexNAc(2)Hex(2)NeuAc(1)	
HexNAc(2)Hex(2)Fuc(1)			

Although various O-glycosites have been identified, their functions remain largely unknown. Similar to N-glycosylation, the functions of O-glycosylation of *S* protein are also very important. More than 60% O-glycosites located close to N-glycosites may suggest the possible complementary functions of O-glycans in immune shielding.^336^ The furin cleavage site is unique to the SARS-CoV-2 S protein compared to SARS-CoV. The O-glycosylation at T678, adjacent to the polybasic furin cleavage site, carries Core-1 and Core-2 structures capped primarily with α2–3 sialic acid, which may suggest that cleavage is potentially regulated by the nearby O-glycans.^332,358^ Mutation of N616 abolished the O-glycosylation at T618 indicates that N-glycosylation at N616 is the prerequisite of N-sequon-related O-glycosylation, which obeys an “O-Follow-N rule”.^80^ T323 and S325 residues are two conserved O-glycosites in the RBD of S1 subunit, which may play important roles in mediating Spike-ACE2 binding.^62,70,328^ Compared with SARS-CoV, S494 is one of the six mutations on the RBD of *S* protein encoded by SARS-CoV-2. Attachment of the O-glycans to S494 can increase the binding affinity of virus to ACE2.^354^ The predicted O-linked glycosylation residues at S673, T678 and S686 are near the RRAR position,^36,337^ implying their potential functions in virus penetration.^332,337,359^

#### E protein

**Fig. 7:**
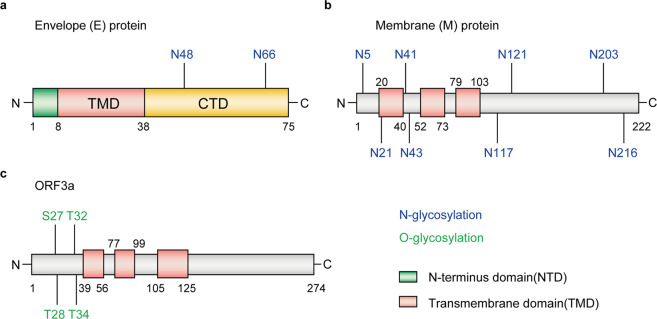
Glycosites of envelope (E) (**a**), membrane (M) (**b**), and ORF3a (**c**) proteins

*E* protein is a small integral membrane protein of 8–12 kDa in SARS-CoV-2,^360^ and functions in viral assembly, release and pathogenesis.^361–363^ It comprises of three domains, including a short hydrophilic N-terminus domain, a hydrophobic transmembrane domain and long hydrophilic C terminal region.^364,365^ Previous studies showed that *E* proteins in many coronaviruses can form pentameric structures exhibiting cation selective channel activity,^366–369^ which is critical for viral infectivity^370,371^ and Ca^2+^ conductivity in the ER-Golgi intermediate compartment.^372^ Based on the sequence prediction, two putative N-linked glycosites may exist in the transmembrane segment of *E* protein at positions N48 and N66^373^ (Fig. 7a). Probably due to the proximity of the residue to the membrane, residue N48 is difficult to be glycosylated.^361^ In contrast, N66 is found to be modified with oligomannose-type glycans.^373^ Mutation of residue N66 can promote the resembling of dimers and trimers of *E* protein which is required for virion assembly, while the monomer may function in disruption of the host secretory pathway.^361^

#### M protein

*M* protein is the most abundant envelope protein of SARS-CoV-2 that contains 222 amino acids.^374,375^ It comprises of three N-terminal transmembrane domains,^374^ and is essential for the assembly of virus particles by interacting with other three structural proteins of SARS-CoV-2.^376–378^ Similar to *E* protein, the glycosylation of M protein has not yet been extensively studied and characterized. In silico computation and simulation has revealed the topology of M proteins from different coronaviruses and predicted eight N-glycosites including N5, N21, N41, N43, N117, N121, N203, and N216 were predicted^379^ (Fig. 7b). The functions of these N-linked glycosylation remain to be studied.

#### ORF3a protein

The non-structural proteins of human coronaviruses are indispensable for viral replication and transcription.^312,380^ *ORF3a* is a non-structural protein of SARS-CoV-2 localized at the surface. It is the largest accessory factor that contains 274 amino acids,^381^ and shows broad functions,^382,383^ such as enhancing viral entry within the host,^381^ regulating the pro-inflammatory cytokine and chemokine production,^384^ participating in ion channel formation as well as modulating release of virus from the host cell.^381^ According to the hydrophobicity analyses and topology studies, there may be four O-linked glycosites at S27, T28, T32 and T34 residues,^385,386^ with higher O-glycosylation occupancy at T28 and T32 residues; N-glycosylation is absent in *ORF3a* protein^382^ (Fig. 7c). The functions of these O-linked glycosylation remain to be investigated.

### Glycosylation of human target protein ACE2

**Fig. 8:**
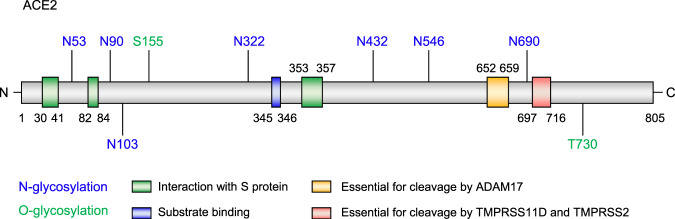
Glycosites of human receptor ACE2

The severity of SARS-CoV-2 infection varies greatly among individuals.^387^ One possible reason may be due to the different expression of SARS-CoV-2 receptor.^40^ ACE2 is the main human receptor of SARS-CoV-2.^388,389^ It is expressed on the membrane of cells located in many organs (such as heart, kidney, and intestines) and is a promising drug target.^390,391^ Besides expression difference, glycosylation on ACE2 also affects the SARS-CoV-2 entry and infectivity.^392^ In the recombinant ACE2 protein from HEK293 cells, 7 N-glycosites and 2 O-glycosites have been identified (Fig. 8). The majority of glycans at N53, N90, N103, N322, N432, N546, and N690 residues of ACE2 are of complex-type, always with >75% occupancy, and the sialic acid linkage always exist in the glycans.^36,40,345^ The sialic acid was previously identified to serve as an attachment factor for a number of coronaviruses including MERS-CoV,^393^ transmissible gastroenteritis virus,^394^ human coronavirus (HCoV)-OC43,^395^ and HCoV-HKU1.^396,397^ The sialic acids present on ACE2 substantially block infection of SARS-CoV; however, the block effect is much smaller in the case of SARS-CoV-2.^398^ In particular, N-glycosylation at residues N90, N322, and N546 of ACE2 play critical roles in the binding of ACE2 with RBD of *S* protein. Mutation of N90 residue increases the binding affinity to *S* protein,^399^ indicating N90 glycosylation can protect host cells against viral infection.^400^ Atomistic molecular dynamics (MD) simulations show that N322 glycan binds to the core region of RBD of *S* protein from amino acid 369 to 378.^40,400^ The interaction between RBD and N322 residue of ACE2 is much stronger than that between RBD and N90 residue of ACE2. Besides, the antibody (CR3022) obtained from SARS-CoV infected patients has a binding site that overlaps remarkably with that of the N322 glycan, suggesting the N322 glycosylation may affect viral infection.^400^ Moreover, MD simulations show that N546 residue involves in the glycan-glycan interactions with *S* protein at N74 and N165.^40^ Of the two identified O-glycosites of ACE2, the stoichiometry of glycosylation at S155 is extremely low, and the function remains elusive.^40^ T730 residue is distal to the binding interface between *S* protein and ACE2, and the Core-1 mucin type O-glycan GalNAcGalNeuAc_2_ is the predominant glycan on it. It is speculated that the massive hydrophilic glycosylation at T730 in the juxtamembrane region outside the cell membrane may affect the dimerization and the presentation of ACE2 on the cell surface.^345^

### Therapeutic strategies for COVID-19 associated with glycosylation

As of 5 April 2021, there are 216 vaccines and 506 therapeutic drugs at different development stages for COVID-19. Among them, 92 vaccines and 419 therapeutic drugs are undergoing clinical trials, and 122 vaccines and 87 therapeutic drugs are in preclinical development (https://biorender.com/covid-vaccine-tracker). Influence of site- and structure-specific glycosylation on infectivity and immune escape is one of the key factors for vaccine development.^31,41,306^ The efficacy of some vaccines and therapeutic drugs may be closely associated with glycosylation (Table 4).

**Table 4:** Drug candidates for SARS-CoV-2 prevention and treatment

Categories	Class	Drug name	Associated glycans or targets	Description	Refs.
Prevention	Natural antibody	GalNAcalpha1-Ser	GalNAc-Ser/Thr (Tn antigen)	Preclinical stage	^414^
		(Gal)1 (GalNAc)1	Gal-GalNAc-Ser/Thr (T antigen)	Preclinical stage	^412^
		FDG-reactive antibodies	Man9-GlcNAc2-Asn	Preclinical stage	^415^
	Vaccine	(GlcNAc)4 (Man)3	GlcNAc(2-4)-Man3-GlcNAc2-Asn	PubChem CID: 10148744	^70,434^
		Man5(GlcNAc)2Asn	Man5-GlcNAc2-Asn	PubChem CID: 25229604	^40,329^
Treatment	Neutralizing antibody	VIR-7831	Glycans at N343	Phase II & NCT04779879	^408^
		VIR-7832	Glycans at N343	Phase I/II & NCT04746183	^408^
		S309	Glycans at N343	Preclinical stage	^406^
	Lectin	FRIL	Complex-type N-glycans	Preclinical stage	^438^
		Griffithsin	Oligosaccharides	Phase I & NCT02875119	^441^
		Clec4g	Complex-type N-glycans	Preclinical stage	^442^
		CD209c	Complex-type N-glycans	Preclinical stage	^442^
		Lentil lectin	oligomannose-type glycans and GlcNAc	Preclinical stage	^443^
	Galectin-3 inhibitor	TD139	N-acetyllactosamine moieties	Phase II/III & NCT04473053	^452,453^
		GB1107	N-acetyllactosamine moieties	Preclinical stage	^450,454^
		Belapectin	N-acetyllactosamine moieties	Phase II & NCT04365868	^455^
	Iminosugars	Castanospermine	α-Glucosidase enzymes	Preclinical stage	^459^
		UV-4	α-Glucosidase enzymes	Preclinical stage	^459^
		Celgosivir	α-Glucosidase enzymes	Phase II & NCT00157534	^459^
		Miglustat	α-Glucosidase enzymes	Preclinical stage	^460^
		Kifunensine	α-Mannosidase enzymes	Preclinical stage	^461^
	Sialidase	DAS181	The sialic acid glycans of virus receptor	Phase 3 & NCT03808922	^463^

*GlcNAc* N-acetylglucosamine, *Gal* galactose, *FDG* Fab-dimerized glycan, *Man* mannose, *Asn* asparagine, *Ser* derine, *Thr* threonine, *FRIL* Flt3 Receptor Interacting Lectin, *UV-4* a monocyclic derivative of DNJ.

#### Neutralizing antibodies

The neutralizing antibodies are one the most important specific defense against viral infection.^348,401,402^ Antibodies that specifically target viral proteins can block the interaction between the virus and the host cell, thereby preventing the virus entry for replication.^403,404^ By high-throughput single-cell sequencing of COVID-19 patients’ B cells, potential SARS-CoV-2 neutralizing antibodies have been found from convalescent patients such BD23-Fab.^348^ Glycosylation at the N165 of *S* protein can facilitate the binding of BD23-Fab to the RBD.^348^ *S* protein has highly conserved glycosylation patterns between SARS-CoV and SARS-CoV-2, the antibodies bound to glycopeptide epitopes of SARS-CoV are critical for the screening of monoclonal antibody (Mab) to treat SARS-CoV-2, such as MAb S309 that has been isolated from SARS-CoV patient targeting an epitope containing a glycan at N343.^334,405,406^ Notably, the antibodies isolated from patients recovering from SARS-CoV, such as the monoclonal antibodies VIR-7831 (Phase II clinical trial), VIR-7832 (Phase I/II clinical trial) and their parent antibody (S309), can also effectively neutralize SARS-CoV-2 in vivo and in vitro.^406–408^ Besides the antibodies from the recovered patients, natural antibodies formed spontaneously without specific immunization may also be very useful for SARS-CoV-2 treatment.^409,410^ GalNAc-O-Ser/Thr (Tn antigen) and Gal-GalNAc-O-Ser/Thr (T antigen) are well-known natural antigens and associated with the pathogenesis of many diseases.^411–413^ Compared to non-infected individuals, the anti-Tn antibodies level in COVID-19 patients are significantly lower, suggesting that natural anti-Tn antibodies may be protective against COVID-19.^414^ In addition, the HIV-1 Env Fab-dimerized glycan (FDG)-reactive antibodies are an anti-glycan antibody that recognize high mannose glycans of SARS-CoV-2, indicating the potential prospects of these natural antibodies in SARS-CoV-2 treatment.^415^

#### Vaccines

Vaccination is the most effective long-term strategy for the prevention and control of COVID-19.^6,416^ Vaccines, such as inactivated vaccines,^417–419^ DNA plasmid vaccines,^420,421^ adenovirus-vectored vaccines,^422,423^ RNA vaccines,^424,425^ protein subunit vaccines,^333,426^ and virus-like particle vaccines,^427,428^ have been developed. In the protein subunit vaccines, the RBD of *S* glycoprotein is an ideal immunogen.^333,429,430^ Because of the existence of glycosites in the immunogenic epitope of the virus, the immunogenic epitopes masked by glycosylation may not be recognized by the host, thus leading to immune escape of the virus.^431^ By mapping the glycosites on the complex structure of the RBD bound to ACE2, it is found that most glycosites are located in the RBD core subdomain and distant from the bound ACE2, indicating that glycans on RBD may not affect receptor recognition and/or binding.^333^ In addition, the viral glycans are also important immunogens.^432,433^ The complex N-glycans such as GlcNAc2-4-Man3-GlcNAc2-Asn in N74, N149, N282, N801, N1074, and N1098 of *S* protein,^70,434,435^ as well as oligomannose-type glycan Man5-GlcNAc2 in N234 may be suitable immunogens for developing vaccines.^40,70,329,434,435^

#### Other drugs

Lectins are carbohydrate-binding proteins binding to sugar groups, and have potent antiviral properties through preventing the attachment of virus to host cell.^59,436,437^ FRIL is a lectin isolated from hyacinth beans and serves as an antiviral agent by blocking the complex-type N-glycans against SARS-CoV-2.^438^ Griffithsin, a red-alga-derived lectin, is in phase I clinical trial for the treatment HIV infection and also is promising for the treatment of COVID-19 by binding to the oligosaccharides on the surface of viral glycoproteins.^439–441^ Other lectins such as Clec4g and CD209c can also bind to the N-glycans of *S* protein and interfere the Spike-ACE2 interaction and reduce SARS-CoV-2 infection.^442^ Notably, Lentil lectin derived from Lens culinaris can bind specifically to oligomannose-type glycans and GlcNAc at the non-reducing end terminus of *S* protein, thus block the binding of ACE2 to S trimer, showing the strongly inhibit infection of SARS-COV-2, including epidemic variants B.1.1.7, B.1.351, and P.1.^443^

The major cause of death by SARS-CoV-2 refers to the “cytokine storm”,^384,444–446^ which is featured as excess release of inflammatory cytokines, such as interleukin (IL)-1, tumor necrosis factor α (TNF-α), and IL-6.^447^ Galectin-3 (Gal-3), a member of β-galactoside-binding lectins that preferentially binds to N-acetyllactosamine moieties on glycoconjugates, showed a dramatic increase with cytokine storm.^448,449^ Inhibition of Gal-3 can reduce the releases of IL-1, TNF-α, and IL-6 from macrophages, suggesting Gal-3 inhibitor as a promising agent for COVID-19 treatment.^450,451^ Currently, the Gal-3 inhibitor TD139 is undergoing clinical trials for the treatment of COVID-19,^452,453^ and other Gal-3 inhibitor such as GB1107,^450,454^ belapectin (also called GR-MD-02) are under investigation.^455^

Iminosugars, also called iminosaccharides, are the analogs of common sugars where an oxygen atom is replaced by a nitrogen atom in the ring of the structure.^456^ They are known to interfere with the N-linked glycosylation by inhibiting the α-glucosidase I and II enzymes on the ER,^457,458^ thus affecting the interaction between viral glycoproteins and host receptor. Iminosugars such as Celgosivir, Castanospermine and the monocyclic UV-4 have been reported to prevent SARS-CoV-2-induced cell death and reduce viral replication,^459^ while Miglustat can lead to a dramatically decrease of the viral Spike protein of SARS-CoV-2.^460^ Other potential inhibitors with similar structures such as α-mannosidase inhibitors Kifunensine also show similar roles in reducing SARS-CoV-2 entry.^36,461^

DAS181 is a kind of inhaled bacterial sialidase that functions by removing sialic acid from the surface of epithelial cells, thus preventing attachment and subsequent infection by respiratory viruses.^462,463^ The sialic acid linkage always existed in the glycans of ACE2,^40,345,392^ suggesting the potential therapeutic effect of DAS181 in COVID-19 treatment. Currently, DAS181 is in phase III clinic trial for patients with severe COVID-19.

### Perspectives

It is well-known that virus may alter the glycan coat on the viral surface to enhance the infectivity and affect immune recognition.^50,464^ With the rapid development of techniques for characterizing the glycans and the glycoproteins,^219,289^ the biological functions and significance of glycans and glycoproteins of virus are disclosed, which broads the understanding of virus biology.^38,45,464^ As described above, both SARS-CoV-2 proteins (especially *S* and *N* proteins) and their receptor (ACE2) are densely glycosylated. The glycan masses on *S* protein, *N* protein and ACE2 are about 80 kDa,^333^ 13 kDa,^465^ and 30 kDa,^466^ respectively; the average mass of a single glycan is about 4 kDa, indicating that these proteins are glycosylated simultaneously on multiple sites, although other modifications, such as phosphorylation,^465^ may also contribute to the extra masses on the basis of the protein sequence. Characterization of glycosylation at the intact N-glycopeptide level with the assistance of state-of-the-art enrichment will deliver comprehensive glycosylation information (glycosite, glycan composition and structure) for single sites,^289^ the cross-talk between different glycosites as well as other PTMs previously missed.^80,467^ Adoption of protein enzymes (such as Glu-C, Asp-N other than trypsin or chymotrypsin) cutting less frequently occurring amino acids to produce larger and longer peptides,^468^ or no enzyme at all (i.e, the top-down method) may be an optional choice.^469^ However, delicate selective dissociation of peptide backbones and glycan moieties as well as versatile bioinformatics tools supporting interpretation of multiple modifications at a time needs to be developed in the future.

The evolution of SARS-CoV-2 is fast within the human population by gaining fitness-enhancing mutations, which may alter viral infectivity and disease severity, and escape the host immunity even in individuals who have been vaccinated. For example, mutation of D to G at the residue 614 (D614G) of *S* protein moderately increases the infectivity and transmissibility.^331,470–472^ Following the D614G mutation, N439K and Y453F mutations within the RBM of *S* protein appears in SARS-CoV-2 variants. These mutations not only enhance the binding affinity for the ACE2 receptor, but also reduce the therapeutic efficacy of neutralizing antibodies.^473,474^ SARS-CoV-2 Delta variant, also known as lineage B.1.617.2, is a variant of lineage B.1.617 of SARS-CoV-2. It has three mutations on *S* protein including T478K, P681R and L452R, which dramatically increases transmission and leads to antibody escape.^475–477^ However, despite many SARS-CoV-2 variants appear, whether the mutations of SARS-CoV-2 variants would affect the glycosylation profile of SARS-CoV-2 is still less understood. Given the critical roles of glycosylation in host recognition, penetration, binding, recycling and pathogenesis, uncovering the glycosylome of SARS-CoV-2 variants may help to increase the understanding of viral biology and develop more effective vaccines and drugs for SARS-CoV-2 variants.

## Data Availability

No additional data are included.
